# Supplementary material for: The Systems Biology Research Tool: evolvable open-source software
Source: BMC Syst Biol. 2008 Jun 29;2:55. doi: 10.1186/1752-0509-2-55 (PMC2446383; doi:10.1186/1752-0509-2-55)
Supplement: Additional file 1 — SBRT Archive. An archive of the current version of the Systems Biology Research Tool. [file 1752-0509-2-55-S1.zip › sbrt-1.4.0/doc/developers_guide/api/sbrt/kernel/math/AbstractLinearComb.html]

AbstractLinearComb


|  |  |  |
| --- | --- | --- |
| **Overview** \| **Package** \| **Class** \| **Use** \| **Tree** \| **Deprecated** \| **Index** \| **Help** | |  |
| PREV CLASS   **NEXT CLASS** | **FRAMES**    **NO FRAMES**     **All Classes** |  |
| SUMMARY: NESTED \| FIELD \| CONSTR \| METHOD | DETAIL: FIELD \| CONSTR \| METHOD |  |


---


## sbrt.kernel.math Class AbstractLinearComb<V>

```
java.lang.Object
  sbrt.kernel.math.AbstractPolynomial<V>
      sbrt.kernel.math.AbstractLinearComb<V>
```

**Type Parameters:**: `V` - the variable type.

**All Implemented Interfaces:**: java.lang.Iterable<LinearComb.Term<V>>, LinearComb<V>, MathExpr<V>, Polynomial<V>

**Direct Known Subclasses:**: HashLinearComb

---

``` public abstract class AbstractLinearComb<V> extends AbstractPolynomial<V> implements LinearComb<V> ```

This abstract class is a skeleton implementation of `LinearComb`.

**Author:**
:   This class was written and documented by Jeremiah Wright
    while in the Wagner lab.

---

| **Nested Class Summary** | |
| --- | --- |
| `static class` | `AbstractLinearComb.Term<V>`             This class is a skeleton implementation of `LinearComb.Term`. |


**Constructor Summary**

`AbstractLinearComb(double constant)`
  
          Constructs a new linear combination with the provided constant.

`AbstractLinearComb(double constant,
double zeroCutoff)`
  
          Constructs a new linear combination with the provided constant and
zero cutoff.


| **Method Summary** | |
| --- | --- |
| `boolean` | `equals(java.lang.Object obj)`             Indicates if the provided object is equal to this linear combination. |
| `java.util.List<LinearComb.Term<V>>` | `getTermList()`             Returns the terms of this linear combination as list. |
| `double` | `getZeroCutoff()`             Returns the current zero cutoff. |
| `java.util.Iterator<LinearComb.Term<V>>` | `iterator()`             Returns an iterator over the terms of this linear combination. |

**Methods inherited from class sbrt.kernel.math.AbstractPolynomial**

`checkConstant, getConstant, hashCode, toString`

**Methods inherited from class java.lang.Object**

`clone, finalize, getClass, notify, notifyAll, wait, wait, wait`

**Methods inherited from interface sbrt.kernel.math.LinearComb**

`add, add, getCoefficient, getTermArray, hashCode, multiply, plugIn, plugIn, plugIn, plugIn, plugInNumbers, solveFor, toString`

**Methods inherited from interface sbrt.kernel.math.Polynomial**

`getConstant, terms`

**Methods inherited from interface sbrt.kernel.math.MathExpr**

`getVariables, hasAllVariables, hasVariable, hasVariables, variables`

**Constructor Detail**

### AbstractLinearComb

```
public AbstractLinearComb(double constant,
                          double zeroCutoff)
```

:   Constructs a new linear combination with the provided constant and
    zero cutoff.

    **Parameters:**: `constant` - the constant of this linear combination.: `zeroCutoff` - the value used to decide if coefficients or constants are close enough to zero to be considered equal to zero. **Throws:**: `java.lang.IllegalArgumentException` - if a provided value is either infinite or NaN; or if the zero cutoff is negative. **See Also:**: `getZeroCutoff()`

---


### AbstractLinearComb

```
public AbstractLinearComb(double constant)
```

:   Constructs a new linear combination with the provided constant. The
    zero cutoff is set to 0d.

    **Parameters:**: `constant` - the constant of this linear combination.


**Method Detail**

### getZeroCutoff

```
public double getZeroCutoff()
```

:   Returns the current zero cutoff.

    The zero cutoff is a value used to decide if coefficients are close
    enough to zero to be considered equal to zero. In other words, the
    absolute value of the coefficient of every term of this linear
    combination will be greater than the zero cutoff.

    :   **Specified by:**: `getZeroCutoff` in interface `LinearComb<V>`
    :   **Returns:**: the zero cutoff.

---


### getTermList

```
public java.util.List<LinearComb.Term<V>> getTermList()
```

:   Returns the terms of this linear combination as list.

    :   **Specified by:**: `getTermList` in interface `LinearComb<V>`
    :   **Returns:**: the terms of this linear combination as list.

---


### iterator

```
public java.util.Iterator<LinearComb.Term<V>> iterator()
```

:   Returns an iterator over the terms of this linear combination.

    :   **Specified by:**: `iterator` in interface `java.lang.Iterable<LinearComb.Term<V>>` **Specified by:**: `iterator` in interface `LinearComb<V>`
    :   **Returns:**: an iterator over the terms of this linear combination.

---


### equals

```
public boolean equals(java.lang.Object obj)
```

:   Indicates if the provided object is equal to this
    linear combination.

    For two linear combinations to be equal, they must contain
    the same numerical constant and terms.

    :   **Specified by:**: `equals` in interface `LinearComb<V>` **Specified by:**: `equals` in interface `MathExpr<V>` **Specified by:**: `equals` in interface `Polynomial<V>` **Overrides:**: `equals` in class `java.lang.Object`
    :   **Parameters:**: `obj` - the object to be tested for equality with this linear combination. **Returns:**: `true` if this linear combination equals the provided object; `false` otherwise. **See Also:**: `AbstractLinearComb.Term.equals(Object)`


---


|  |  |  |
| --- | --- | --- |
| **Overview** \| **Package** \| **Class** \| **Use** \| **Tree** \| **Deprecated** \| **Index** \| **Help** | |  |
| PREV CLASS   **NEXT CLASS** | **FRAMES**    **NO FRAMES**     **All Classes** |  |
| SUMMARY: NESTED \| FIELD \| CONSTR \| METHOD | DETAIL: FIELD \| CONSTR \| METHOD |  |


---
